# Diagnosis and Management of a Unilateral Posterior Open Bite Using a Temporary Anchorage Device (TAD): Case Report and Review of the Literature

**DOI:** 10.1155/2020/9814949

**Published:** 2020-02-01

**Authors:** Bandar Alyami

**Affiliations:** Department of Preventive Dentistry, Faculty of Dentistry, Najran University, Najran, Saudi Arabia

## Abstract

This report describes the diagnosis and successful treatment of a unilateral posterior open bite (POB) in a 15-year-old Caucasian boy. Simple mechanics were used to rule out ankylosis of left posterior teeth as the etiological factor of the POB. Thereafter, the same mechanics were continued to expand the unilateral constricted maxilla, to create a space, and to close POB. Sectional biomechanics were applied to avoid undesirable tooth movements. Then, continuous arch wires were employed to coordinate arches and to achieve treatment objectives.

## 1. Introduction

A posterior open bite (POB) is defined as failure of a number of teeth in either or both opposing buccal segments to reach occlusion while there is an incisal contact [1]. Two scenarios have been described in POB: the first one is when only the anterior teeth are touching with no single posterior teeth touching and the second one is when the anterior teeth and some of the posterior teeth are touching. Generally, an open bite is present in about 25%-38% of patients treated orthodontically [2], and the etiology is multifactorial from interaction between genetic and environmental factors [1]. POB is a rare condition and can be attributed to conditions like interpositional tongue habit, digit habit (sucking or chewing), mouth breathing, adenoid hypertrophy, syndromes, and partial eruption of the first premolars [3]. Other causes of open bite include obstacles in the path of eruption such as supernumerary teeth and nonresorbing deciduous roots [3–5].

Patients with POB can have functional and psychological problems [1]. The functional problems include defective speech, mastication challenges, and problems with deglutition resulting in child's impaired development [1]. Treatment of POB and the retention of the posterior teeth in occlusion can be two of the most difficult problems an orthodontist can face. When this condition occurs unilaterally, it requires complex management strategies [6]. Several treatment options have been reported for the management of POB. These methods include habit breakers, myofunctional appliances in the growing child, orthodontic removable appliances, fixed orthodontic appliances including temporary anchorage device (TAD), and orthognathic surgery for a skeletal open bite [7–12]. In this case report, the TAD was used to confirm if the unilateral POB was a result of ankylosis and thereafter used to successfully treat the condition. A literature search has not highlighted this concept in the management of POB.

## 2. Case Report

**Figure 1:**
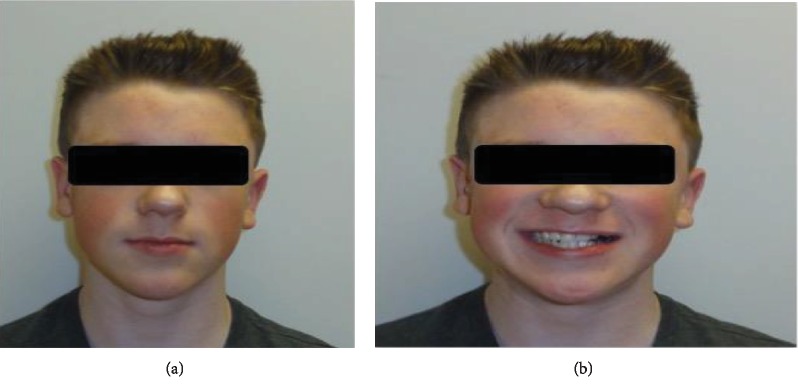
(a) Pretreatment frontal view of the patient. (b) Pretreatment frontal smile view of the patient.

A 15-year-old Caucasian male presented with the complaints “I cannot bite properly on left side.” His medical, dental, familial, and social history was not contributory. He has no abnormal oral habit. On examination, he has an asymmetrical dolichofacial face (Figure 1(a)), competent lips, an obtuse nasolabial angle, and an orthognathic slightly convex profile. His upper lip was 9 mm behind the E-line, and his lower lip was 4 mm behind the E-line. His maxillary midline was 1 mm to the left of the facial midline, and his mandibular midline is 2 mm to the left of the chin (Figure 1(b)).

**Figure 2:**
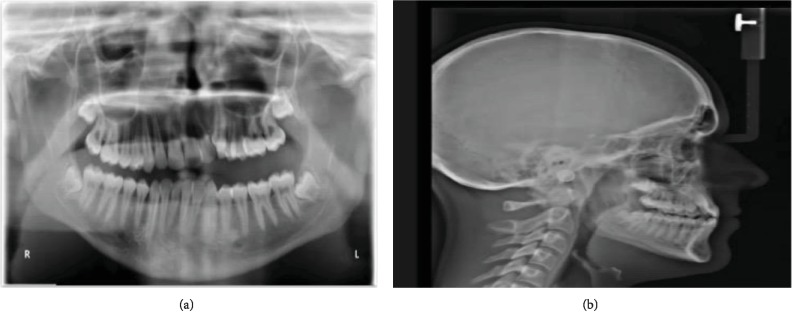
(a) Pretreatment panoramic X-ray showing all teeth present without any pathology and unlevelled upper and lower left posterior segments. (b) Pretreatment cephalometric X-ray of the patient's skeletal class I pattern with an increase in the length of the mandible and maxilla and increased lower facial height.

**Figure 3:**
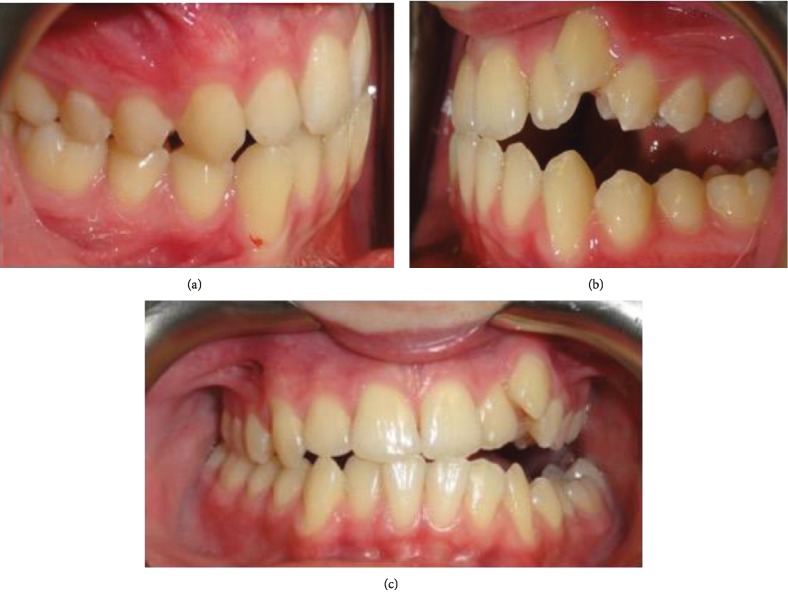
(a) Intraoral lateral view showing the right class III molar and canine relationships. (b) Intraoral lateral view showing the left POB and undetermined molar and canine relationships. (c) Intraoral view showing maxillary crowding with the blocked out upper left canine.

**Figure 4:**
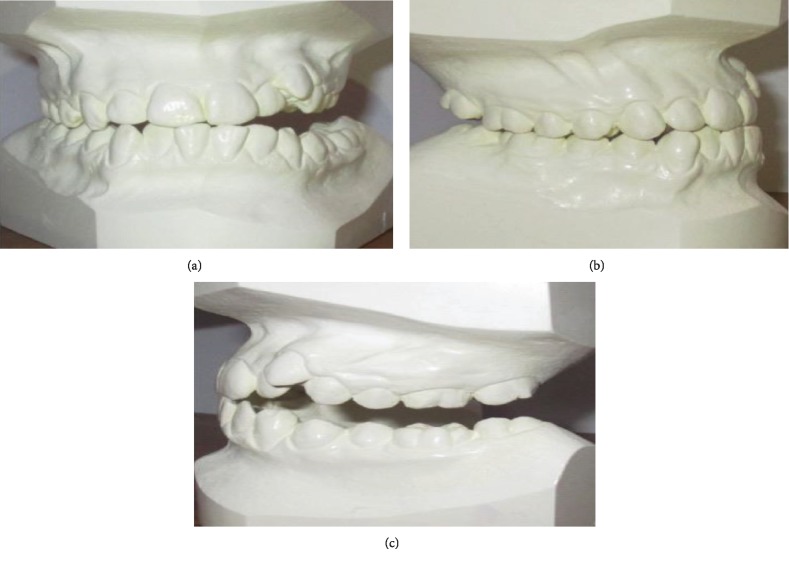
(a) Pretreatment frontal view of the patient's cast. (b) Pretreatment right lateral view of the patient's cast. (c) Pretreatment left lateral view of the patient's cast.

Cephalometric analyses (Table 1) revealed a normodivergent profile.  Figure 2(a) shows a skeletal class I pattern with an increase in the length of the mandible and maxilla and increased lower facial height. Panoramic X-ray showed that all teeth were present with no pathology seen (Figure 2(b)). He has a dentally class III molar and canine relationship on the right while the left molar and canine relationships cannot be determined (Figures 3(a) and 3(b)). In addition, there were 6.71 mm and 4 mm of crowding in the upper and lower arches, respectively. He also has an edge-to-edge incisor relationship (Figure 3(c)), a posterior open bite on the left side, and a curve of Spee of 3.5 mm. Figures 4(a)–4(c) show pretreatment cast views of the patient.

## 3. Problem List

The problem list included a unilateral posterior open bite and constricted maxilla on the left side, severe maxillary crowding (blocked out upper left canine) and moderate mandibular crowding, edge-to-edge incisal relationship, deep curve of Spee, class III molar and canine relationship, maxillary midline shift of about 1 mm to the left, and mandibular left midline shift of about 2 mm.

## 4. Treatment Objectives

The objectives of the treatment were to initially find out if etiology of the POB was due to ankylosis (if not, treatment will proceed with the closing of the left posterior open bite), to alleviate crowding and bring the upper left canine to normal position, to improve overbite and overjet, to level the curve of Spee, to achieve a class I molar and canine relationship, and to correct midline shifts in the maxillary and mandibular arches.

## 5. Treatment Plan

The treatment plan included bonding of the upper left first premolar, upper left second premolar, and upper left first molar and placement of a palatal button, a TAD between the lower left second premolar and lower left first molar buccally, a sectional arch wire in the upper left side, and rubber bands from the palatal button to the TAD; use of an advanced arch wire 2 × 4 to correct edge-to-edge anterior bite and bonding of lower arches from the right second molar to the left second molar; and then placement of a sectional arch wire, leveling and aligning of upper and lower arches, interproximal reduction (IPR) of upper and lower teeth, coordination of arches, finishing, detailing, and finally retention.

## 6. Mechanotherapy

The challenge in this case was what kind of mechanics can correct the constricted maxilla, create a space, and extrude teeth to close the POB without any side effect on other teeth that are in a proper position.

**Figure 5:**
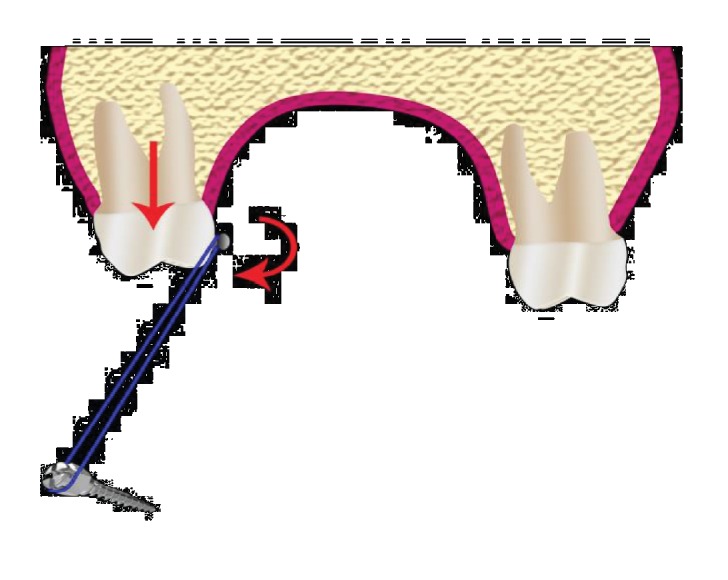
Schematic diagram showing the mechanotherapy principle employed.

The patient was asked to wear the elastic from the palatal button of the upper teeth to the TAD on the lower arch (Figure 5). The force applied from the palatal button was passing away from the center of resistance of the segment which creates a moment leading to buccal tipping and downward movement of the segment. The use of these mechanics allowed us to diagnose any possible presence of ankylosed teeth with no effect on adjacent and/or opposing teeth. After extruding the posterior segment to the same level of adjacent teeth, an advanced arch wire was used to correct the edge-to-edge bite. Then, continuous arch wires were used to coordinate arches.

## 7. Treatment Progress

**Figure 6:**
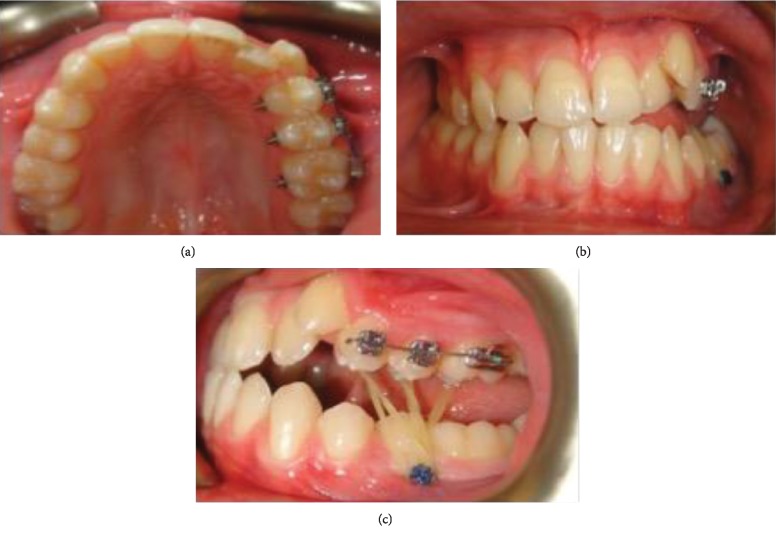
(a) Intraoral radiograph showing palatal buttons. (b) Intraoral radiograph showing TAD between the second premolar and first molar. (c) Intraoral radiograph showing elastics 5/16 4 oz placed from the palatal buttons to the TAD.

After taking initial records, the upper left first premolar, upper left second premolar, and upper left first molar were bonded at the same level to allow the insertion of a 0.018 × 0.025 TMA wire (Figure 6(a)). Thereafter, palatal buttons were bonded and TAD was placed between the lower left second premolar and lower left first molar. The patient was asked to wear elastics size 5/16 4 oz from the palatal buttons to the TAD (Figures 6(b) and 6(c)). After 2 months of treatment, the use of elastics was stopped and the lower left canine, lower left first premolar, lower left second premolar, and lower left first molar were bonded to commence levelling and alignment. The patient was lost to follow-up for 4 months.

Following 5 months of leveling and alignment, the TAD was removed and the upper right central incisor, upper right lateral incisor, upper right first molar, upper left central incisor, and upper left lateral incisor were bonded, and an advancement arch wire size 0.018 SS was placed to correct the overjet.

A lower sectional arch wire was then upgraded from sectional 0.016 NITI to sectional 0.016 × 0.022 NITI. After one month of treatment, the overjet improved and thereafter the posterior left side elastic was placed from the upper lingual button to the lower buccal brackets. In the following month, the lingual button was placed on the lower left first premolar, lower left second premolar, and lower left first molar and 3/16 4 oz elastics were placed. These mechanics were maintained for another 2 months. The remaining upper teeth were bonded for levelling and alignment. IPR was done for the lower right central incisor, lower right lateral incisor, and lower left central incisor and from the upper right second premolar to the upper left second premolar. The upper arch wire was then upgraded to 0.018 × 0.025SS and lower arch wire upgraded to 0.020 SS for another 6 months.

## 8. Result

**Figure 7:**
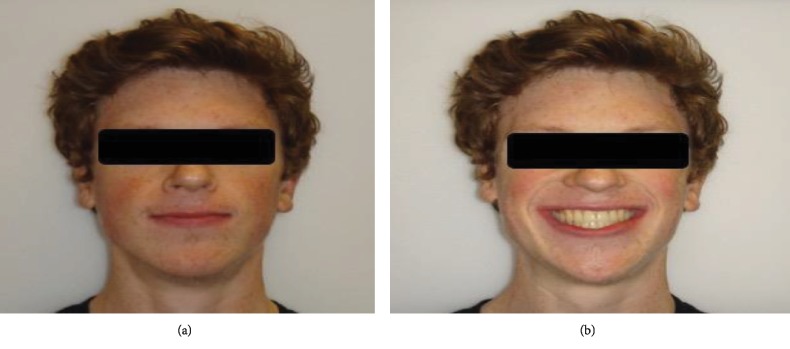
(a) Posttreatment frontal view of the patient. (b) Posttreatment frontal smile view of the patient.

**Figure 8:**
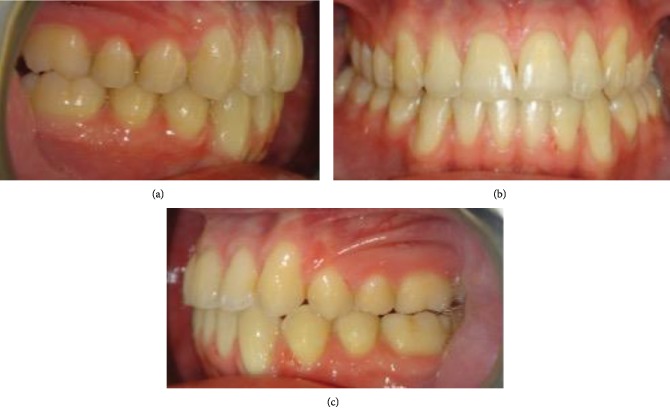
(a) Posttreatment intraoral lateral photograph showing the right class I molar and canine relationship. (b) Posttreatment intraoral photograph showing the corrected midline. (c) Posttreatment intraoral photograph showing the left class I molar and canine relationship and correction of the left POB.

**Figure 9:**
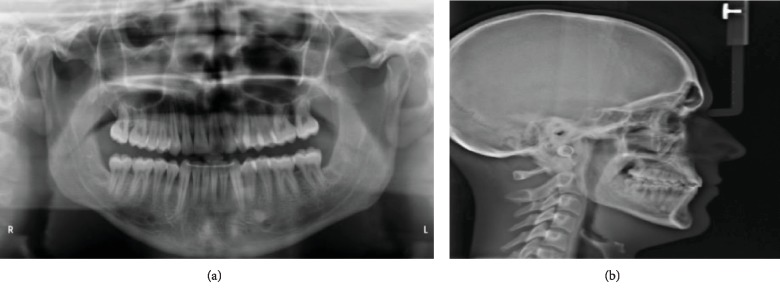
(a) Posttreatment panorama showing levelling of the upper and lower left posterior segments. (b) Posttreatment cephalometric X-ray showing minor skeletal changes with slight changes in the upper and lower incisor inclination.

**Figure 10:**
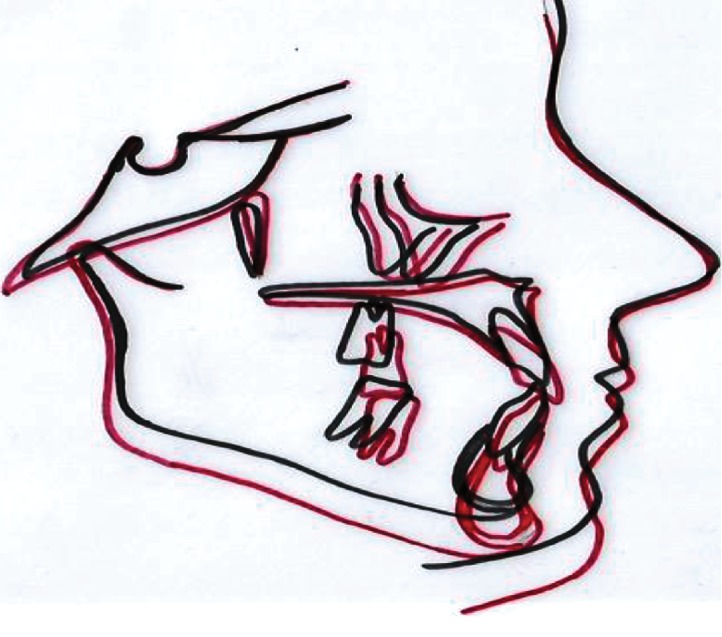
Cephalometric superimpositions showing skeletal changes (black is pretreatment and red is posttreatment).

**Figure 11:**
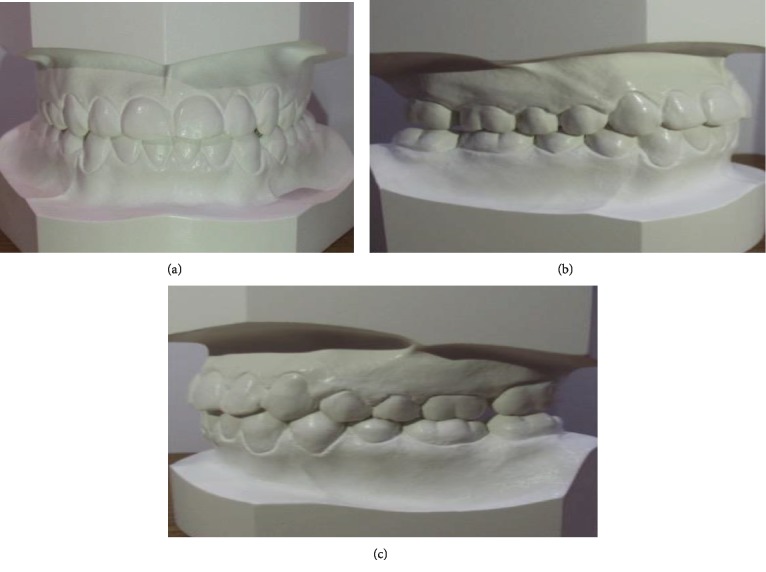
(a) Posttreatment frontal view of the patient's cast. (b) Posttreatment right lateral view of the patient's cast. (c) Posttreatment left lateral view of the patient's cast.

After 18 months of active treatment, the overjet and overbite were within a normal value and the left unilateral open bite and midline shift were corrected (Figures 7(a) and 7(b)). In addition, there were satisfactory leveling and alignment, and alleviation of crowding and a class I molar and canine relationship were achieved (Figures 8(a)–8(c)). Cephalometric X-ray (Figure 9(a)) showed minor skeletal changes posttreatment (Table 1). Panoramic X-ray (Figure 9(b)) showed levelling of the upper and lower left posterior segments posttreatment.  Figure 10 shows cephalometric superimpositions. Figures 11(a)–11(c) show posttreatment casts.

## 9. Retention

Because of the strong cheek muscle observed in this patient which might have been the etiological factor of the POB, modified Hawley's retainer with acrylic part on the buccal side was fabricated to hold the cheek away from the occlusion thereby allowing maximum intercuspation of the posterior segment during the retention period.

## 10. Discussion

Management of open bites is often challenging because of high relapse rate, and patients' cooperation is highly important. When such open bites are skeletal and the patient declines surgery, then an orthodontic option for treatment is opted for which needs more time and patient cooperation [13]. Studies have recommended TADs for the provision of anchorage in the extrusion and intrusion of posterior teeth [7, 10, 14, 15]. This mechanism had been shown to close POB and reduce facial height without any form of surgical intervention [10, 16]. Etiologies of POB are countless, and diagnosis of a particular cause can be challenging. In this report, the TAD was used as both diagnostic and therapeutic devices. Clinically, it is important to differentiate whether the failure of eruption was due to disruption of the eruption mechanism or primary failure [16]. In this case, the patient did not give any history of habit therefore making decision on the mode of intervention difficult. To unravel this impasse, it was decided to place the TAD to see if there will be any form of tooth movement. When movement was observed, the device then proceeded to the treatment phase. If the POB was due to ankylosis, then no tooth movement will be observed and the device should be terminated immediately to prevent complications. In the current report, tooth movement was observed thereby eliminating ankylosis as the etiological factor. A literature search did not report this novel option of the diagnostic effect of TAD.

During the initial active treatment phase, a sectional biomechanics principle was employed to avoid undesirable tooth movement and to directly target the problem site. Undesirable tooth movement during the active treatment phase will result in a side effect of which the solution requests additional treatment time. It may also result in increased biological damage to the dentition and possible root resorption [17]. Sectional biomechanics were introduced by Dr. Charles Burstone in 1962 and consist of a sequence of orthodontic procedures based on the mechanical principles of mechanics [18]. In this case, the dental arch was divided into three major segments, one anterior (incisors and canines) and two posterior (premolars to molars) [19]. The rationale behind this division was to have a better controlled tooth movement than arch wire-guided tooth movement [19]. In the present case report, the left posterior upper and lower segments were focused on the principle of sectional biomechanics. Following the extrusion of the teeth in the upper left and lower left segments, the sectional biomechanics were changed to a continuous arch wire mechanism for levelling and alignment.

In order to correct the edge-to-edge bite, a two-by-four sectional appliance was applied after the correction of the unilateral POB. This fixed orthodontic appliance comprises four brackets bonded onto the four erupted maxillary permanent incisors and two bands cemented or two tubes bonded on the first permanent molars with a continuous arch wire to provide and maintain a good arch form [20]. This fixed appliance allows swift correction of many emerging malocclusions in a distinct short phase of fixed appliance therapy during the early mixed dentition stage [21]. In addition, a two-by-four appliance may also lessen the complexity and interval of any future treatment.

## 11. Conclusion

Diagnosing the etiological factors and treatment of posterior open bite malocclusion is often challenging. Treatment modalities include myofunctional appliances in growing children and surgeries in adults. Minor cases can be managed by fixed orthodontics especially with TADs alongside with some habit-breaking appliances if any form of it was identified. Additional care should be taken while diagnosing and planning treatment for such cases as any mistake in identifying the etiological factor may lead to a poor end result.

## Figures and Tables

**Table 1:** Cephalometric evaluation: normal, pretreatment, and posttreatment.

	Normal values	Pretreatment	Posttreatment
SNA	81 ± 2	84	85.1
SNB	80 ± 2	83.4	83.4
ANB	4 ± 2	1.4	1.7
Convexity	6.8	0.9	1.1
SN-Go Gn	32.9	29.3	28
UFH:LFH, lower (ANS-Gn/N-Gn)	55	62.8	63.3
U1-SN (°)	103.1	106.3	109.3
L1-NB (°)	25.3	25.9	23
Mandibular length (Co-Gn) (mm)	122.1	179.5	181.6
Maxillary length (Co-A) (mm)	90.0	121.5	121.3

## References

[1] Wajid M. A., Chandra P., Kulshrestha R., Singh K., Rastogi R., Umale V. (2018). Open bite malocclusion: an overview. *Journal of Oral Health and Craniofacial Science*.

[2] Espeland L., Dowling P. A., Mobarak K. A., Stenvik A. (2008). Three-year stability of open-bite correction by 1-piece maxillary osteotomy. *American Journal of Orthodontics and Dentofacial Orthopedics*.

[3] Matsumoto M. A. N., Romano F. L., Ferreira J. T. L., Valerio R. A. (2012). Open bite: diagnosis, treatment and stability. *Brazilian Dental Journal*.

[4] Southard T. E., Marshall S. D., Bonner L. L. (2015). Posterior Open Bites. *Orthodontics in the vertical dimension: a case-based review*.

[5] Atobe M., Sekiya T., Tamura K., Hamada Y., Nakamura Y. (2009). Severe lateral open bite caused by multiple ankylosed teeth: a case report. *Oral Surgery, Oral Medicine, Oral Pathology, Oral Radiology, and Endodontology*.

[6] Cafferty J. M., Awadi E. A., O’Connell A. C. (2010). Management of severe posterior open bite due to primary failure of eruption. *European Archives of Paediatric Dentistry*.

[7] Kim Y. H., Han U. K., Lim D. D., Serraon M. L. (2000). Stability of anterior openbite correction with multiloop edgewise archwire therapy: a cephalometric follow up study. *American Journal of Orthodontics and Dentofacial Orthopedics*.

[8] Sugawara J., Baik U. B., Umemori M. (2002). Treatment and posttreatment dentoalveolar changes following intrusion of mandibular molars with application of a skeletal anchorage system (SAS) for open bite correction. *The International Journal of Adult Orthodontics and Orthognathic Surgery*.

[9] Melsen B., McNamara J. A., Hoenie D. C. (1995). The effect of bite-blocks with and without repelling magnets studied histomorphometrically in the rhesus monkey (Macaca mulatta). *American Journal of Orthodontics and Dentofacial Orthopedics*.

[10] Park Y. C., Lee H. A., Choi N. C., Kim D. H. (2008). Open bite correction by intrusion of posterior teeth with miniscrews. *The Angle Orthodontist*.

[11] de Castro Cabrera M., Cabrera C. A. G., de Freitas K. M. S., Janson G., de Freitas M. R. (2010). Lateral open bite: treatment and stability. *American Journal of Orthodontics and Dentofacial Orthopedics*.

[12] Ahn H.-W., Chung K.-R., Kang S.-M., Lin L., Nelson G., Kim S.-H. (2012). Correction of dental class III with posterior open bite by simple biomechanics using an anterior C-tube miniplate. *The Korean Journal of Orthodontics*.

[13] Stuani M. B. S., Stuani A. S., Stuani A. S. (2005). Modified Thurow appliance: A clinical alternative for correcting skeletal open bite. *American Journal of Orthodontics and Dentofacial Orthopedics*.

[14] Moon C. H., Lee J. S., Lee H. S., Choi J. H. (2009). Non-surgical treatment and retention of open bite in adult patients with orthodontic mini-implants. *The Korean Journal of Orthodontics*.

[15] Kim M.-J., Park S.-H., Kim H.-S. (2011). Effects of orthodontic mini-implant position in the dragon helix appliance on tooth displacement and stress distribution: a three-dimensional finite element analysis. *The Korean Journal of Orthodontics*.

[16] Deguchi T., Kurosaka H., Oikawa H. (2011). Comparison of orthodontic treatment outcomes in adults with skeletal open bite between conventional edgewise treatment and implant-anchored orthodontics. *American Journal of Orthodontics and Dentofacial Orthopedics*.

[17] Caldas S. G. F. R., Ribeiro A. A., Simplício H., Machado A. W. (2014). Segmented arch or continuous arch technique? A rational approach. *Dental Press Journal of Orthodontics*.

[18] Burstone C. J. (1962). Rationale of the segmented arch. *American Journal of Orthodontics*.

[19] El-Bialy T. (2013). Segmented and sectional orthodontic technique: review and case report. *Journal of Health Specialties*.

[20] Quinzi V., Ferro R., Rizzo F. A. (2018). The two by four appliance: a nationwide cross-sectional survey. *European Journal of Paediatric Dentistry*.

[21] McKeown H. F., Sandlerd J. (2001). The two by four appliance: a versatile appliance. *Dental Update*.

